# Supplementary figures and images for: Multiomic characterization of disease progression in mice lacking dystrophin
Source: PLoS One. 2023 Mar 31;18(3):e0283869. doi: 10.1371/journal.pone.0283869 (PMC10065259; doi:10.1371/journal.pone.0283869)

WT

*mdx*

*mdx* *utrn*<sup>+/+</sup>

*mdx* *utrn*<sup>+/-</sup>

5X

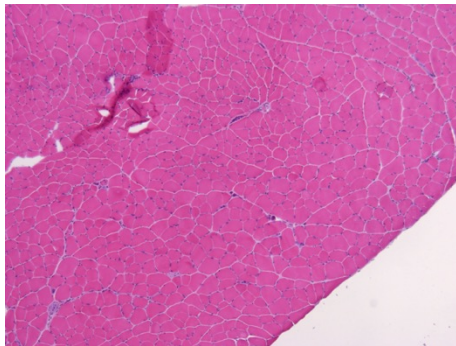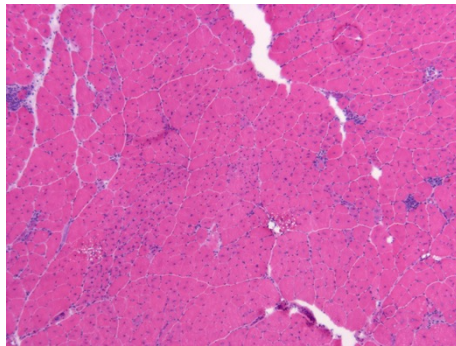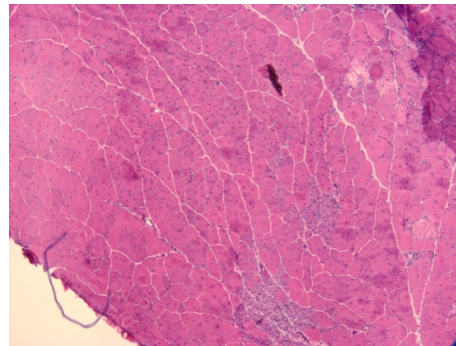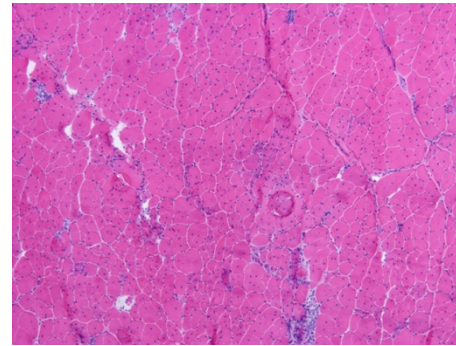

20X

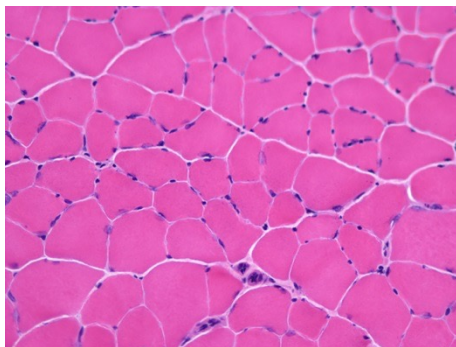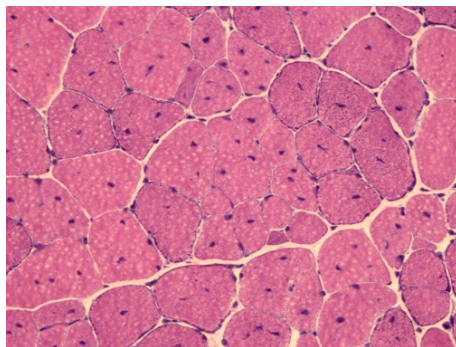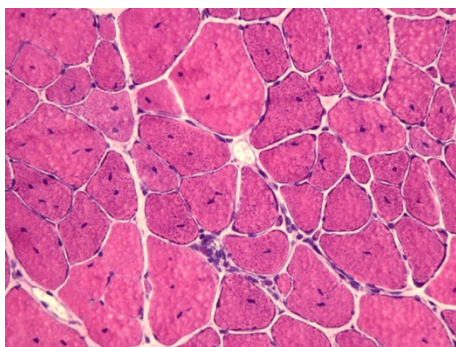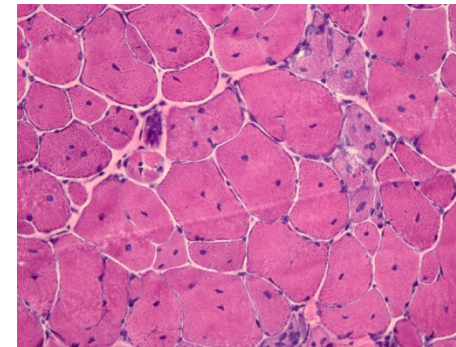

Supplementary Figure 1

Supplement: S1 Fig — Image acquisition was performed at 5X (top panels) and 20X (bottom panel) magnification. (PDF) [file pone.0283869.s001.pdf]

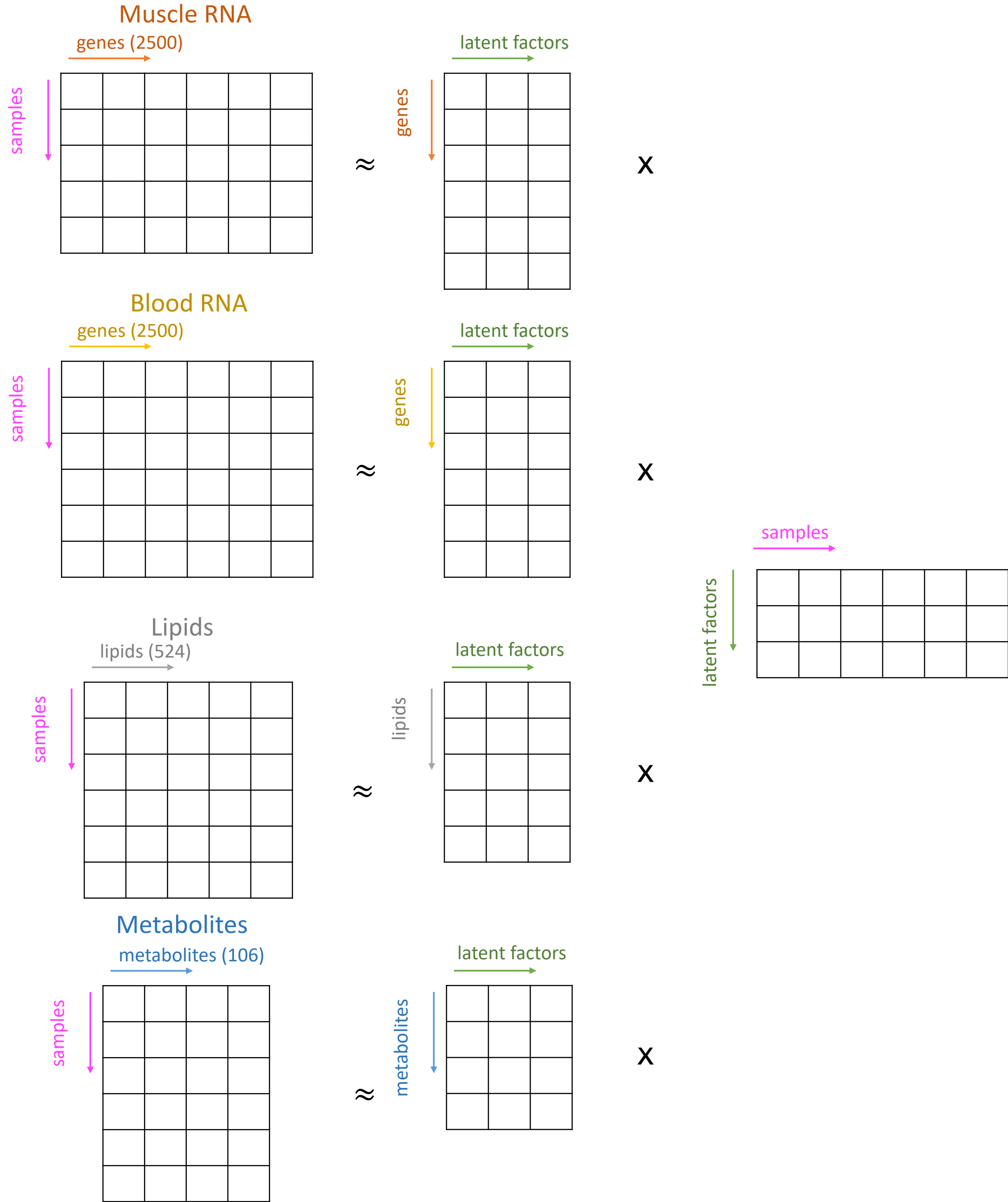

Supplement: S2 Fig — (PDF) [file pone.0283869.s002.pdf]

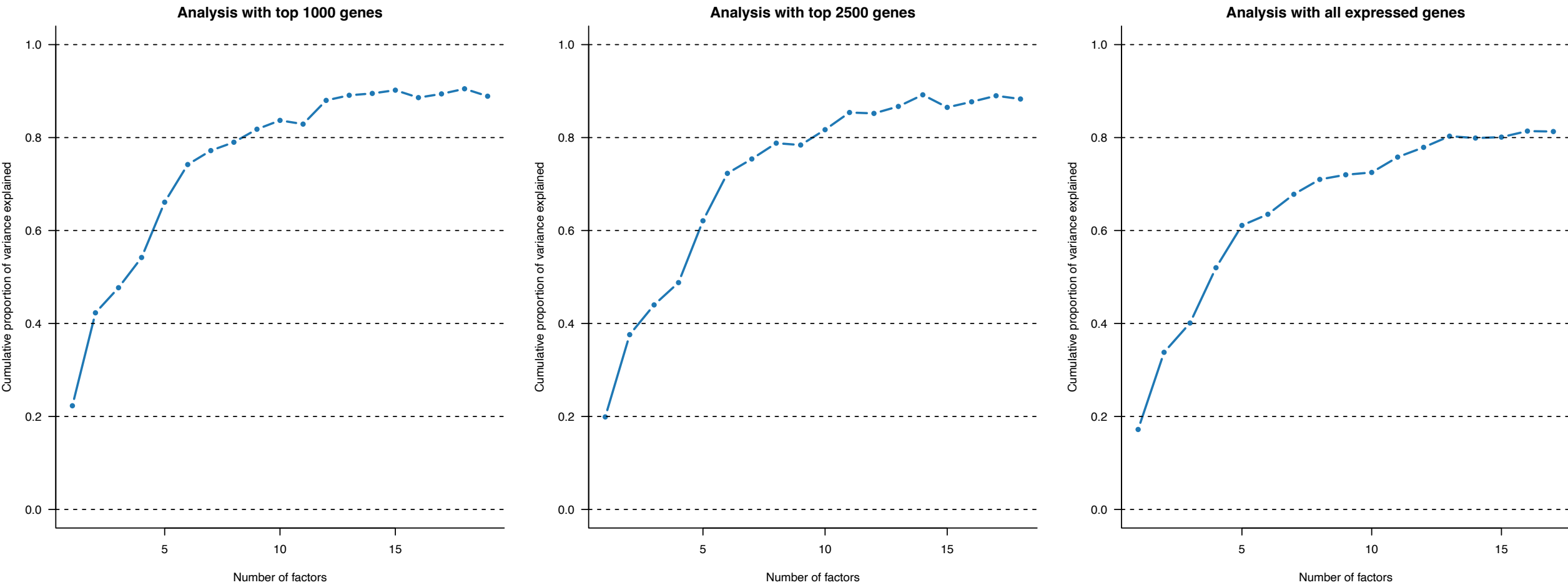

Supplementary Figure 1

Supplement: S3 Fig — Percentage of total variance explained versus number of LFs for MOFA models fitted including the top 1000 (left) or 2500 (center) genes by variance in blood and muscle RNA seq, or all expressed genes (11243 in muscle and 10349 in blood; right). (PDF) [file pone.0283869.s003.pdf]

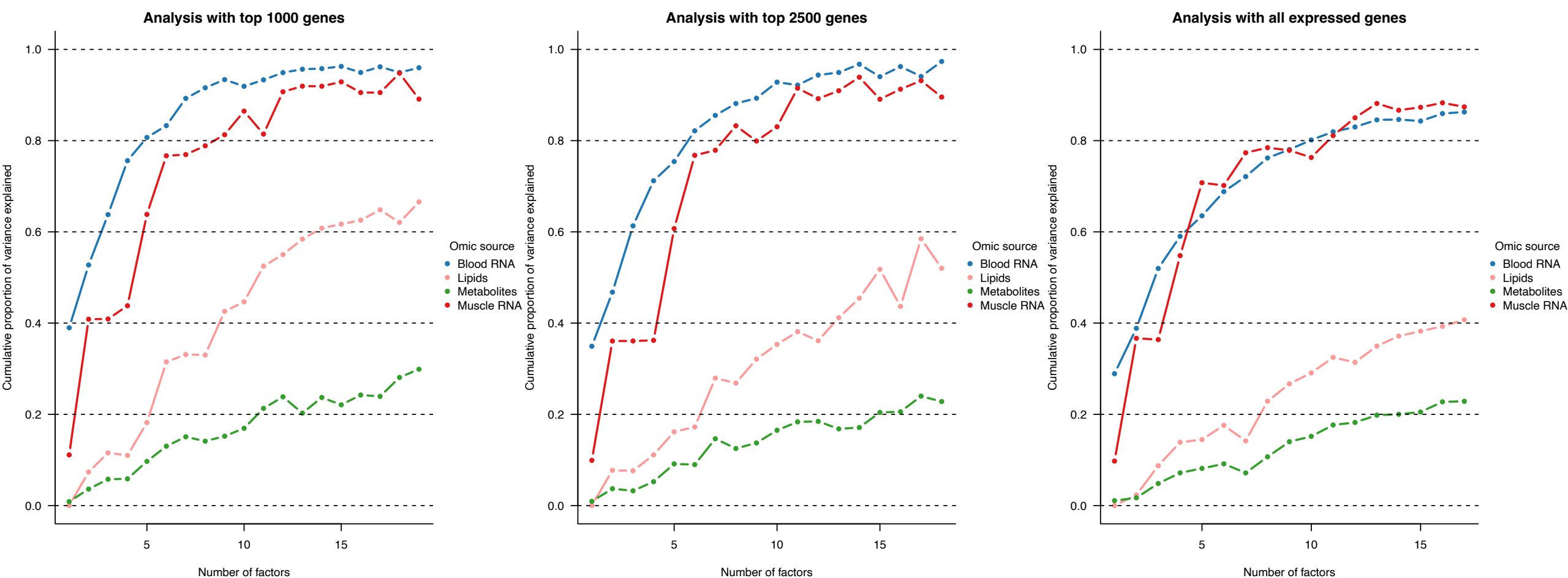

Supplementary Figure 2

Supplement: S4 Fig — Comparison of the percentage of variance explained in the 4 omic views versus number of LFs for MOFA models fitted including the top 1000 (left) or 2500 (center) genes by variance in blood and muscle RNA seq, or all expressed genes (11243 in muscle and 10349 in blood; right). (PDF) [file pone.0283869.s004.pdf]

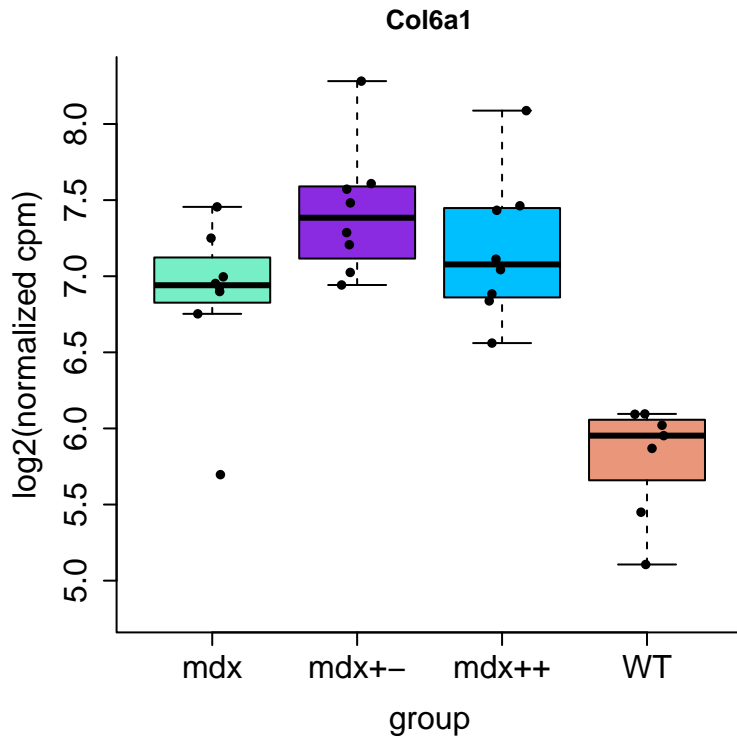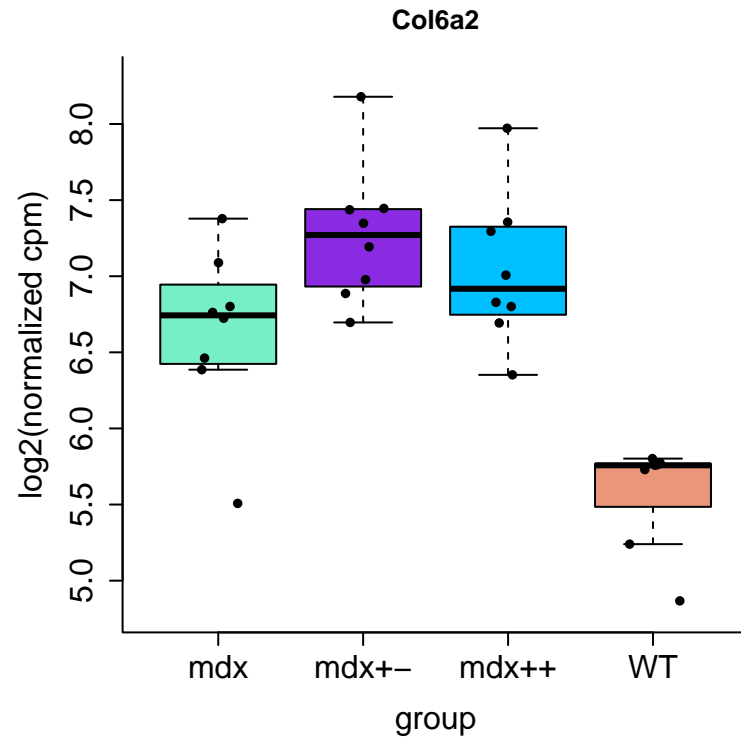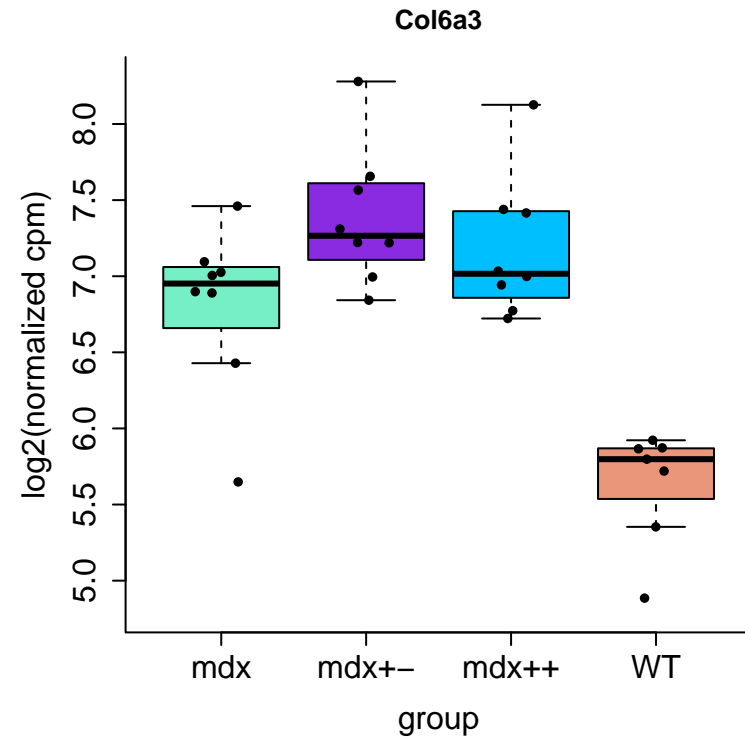

Supplement: S5 Fig — (PDF) [file pone.0283869.s005.pdf]

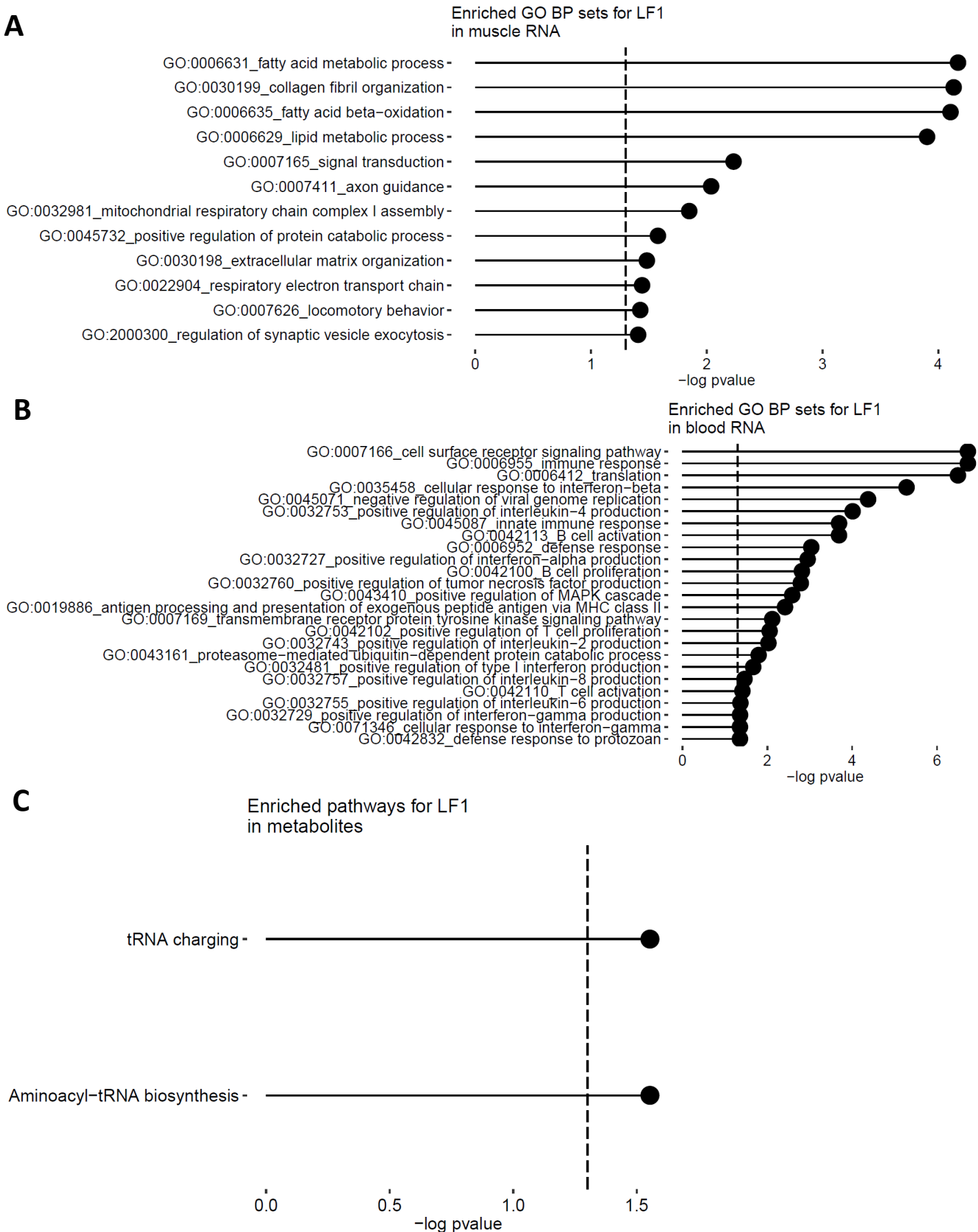

Supplementary Figure 4

Supplement: S6 Fig — Principal component gene set enrichment analysis of LF1 in blood RNA (panel A), muscle RNA (panel B) and metabolites (panel C). (PDF) [file pone.0283869.s006.pdf]

A

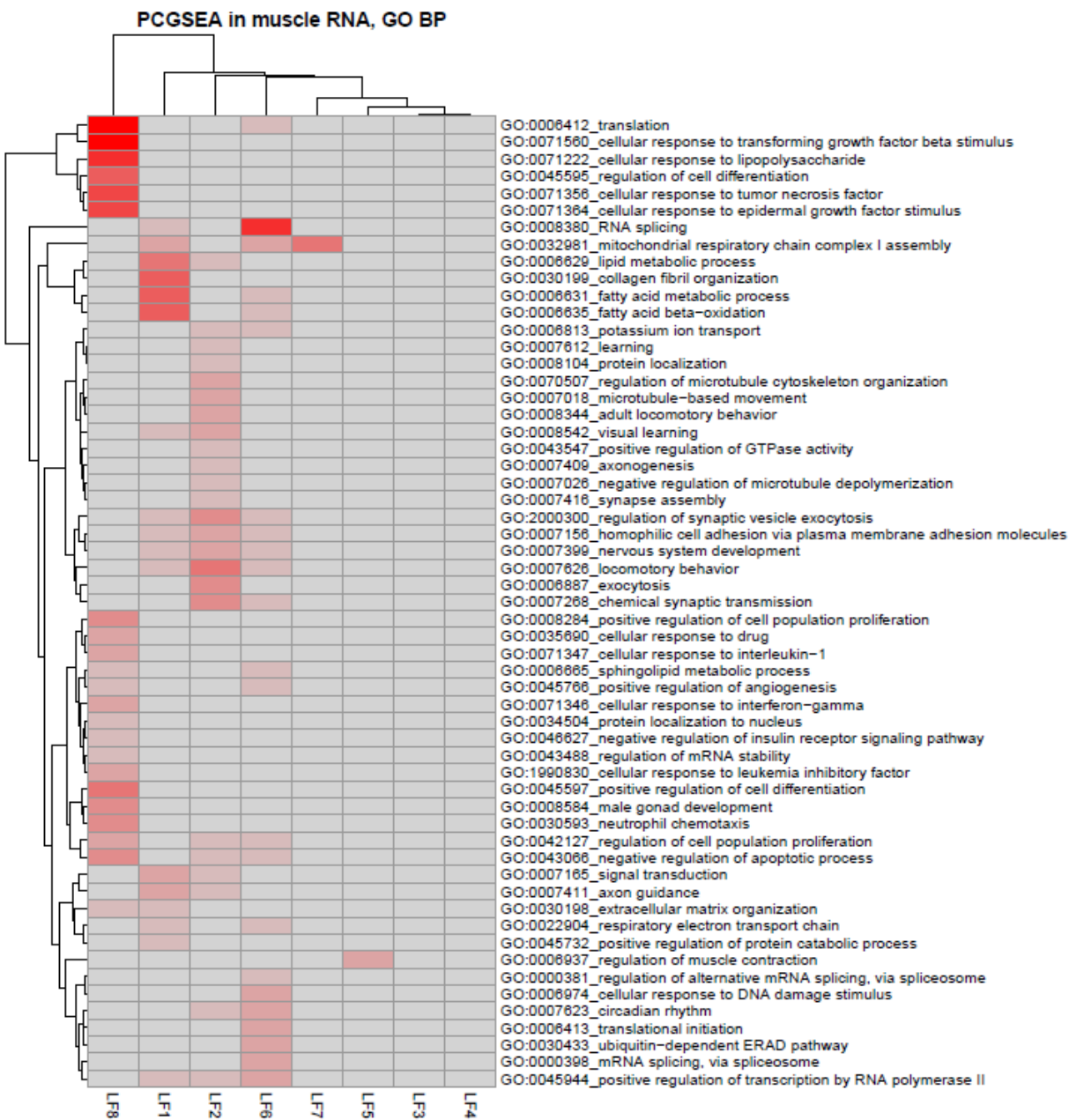

B

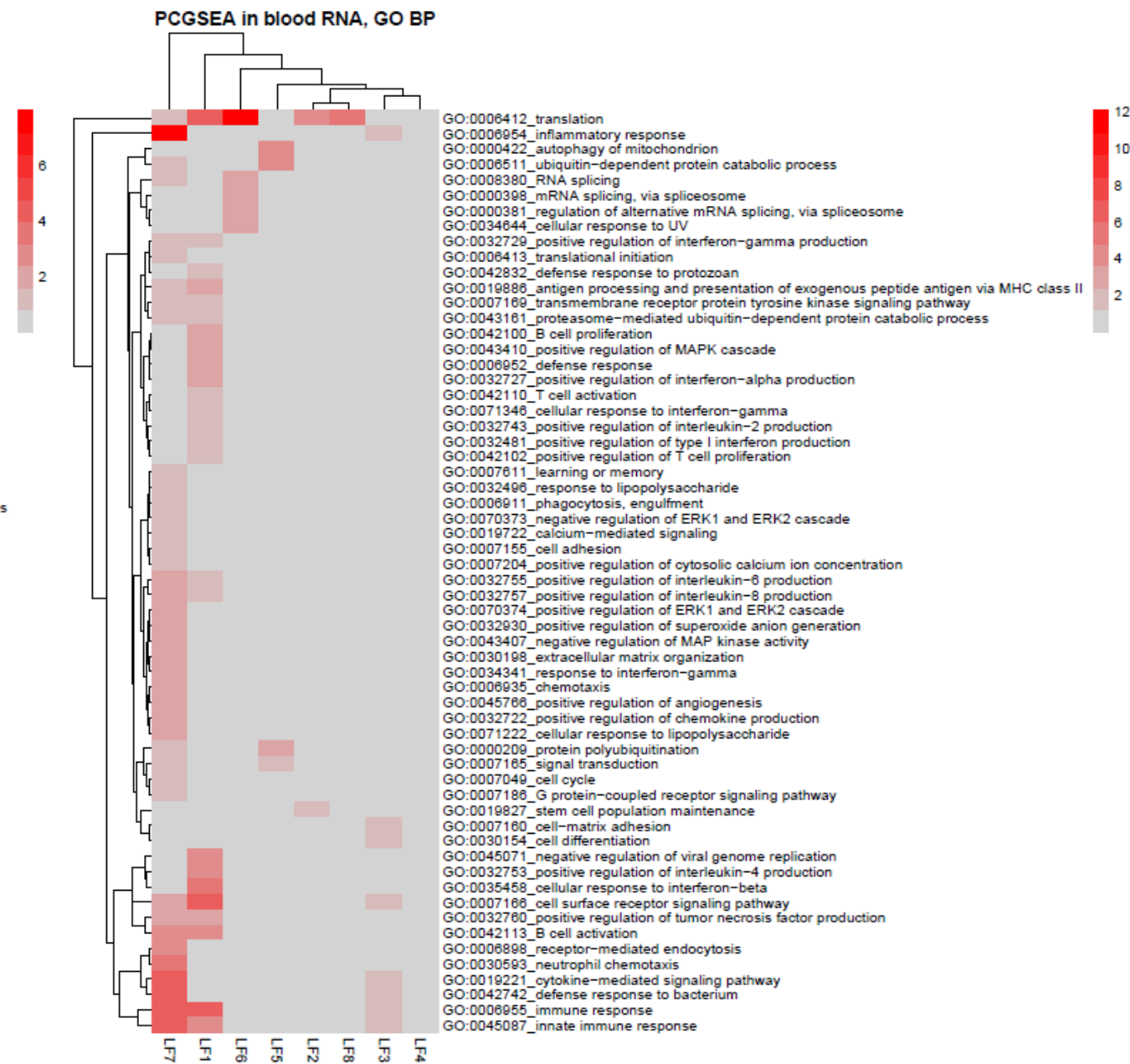

Supplementary Figure 5

Supplement: S7 Fig — Heatmap of the log-p values of gene sets by factor obtained from the PCGSEA of the GO BP gene ontology in muscle RNA (panel A) and blood RNA (panel B). (PDF) [file pone.0283869.s007.pdf]

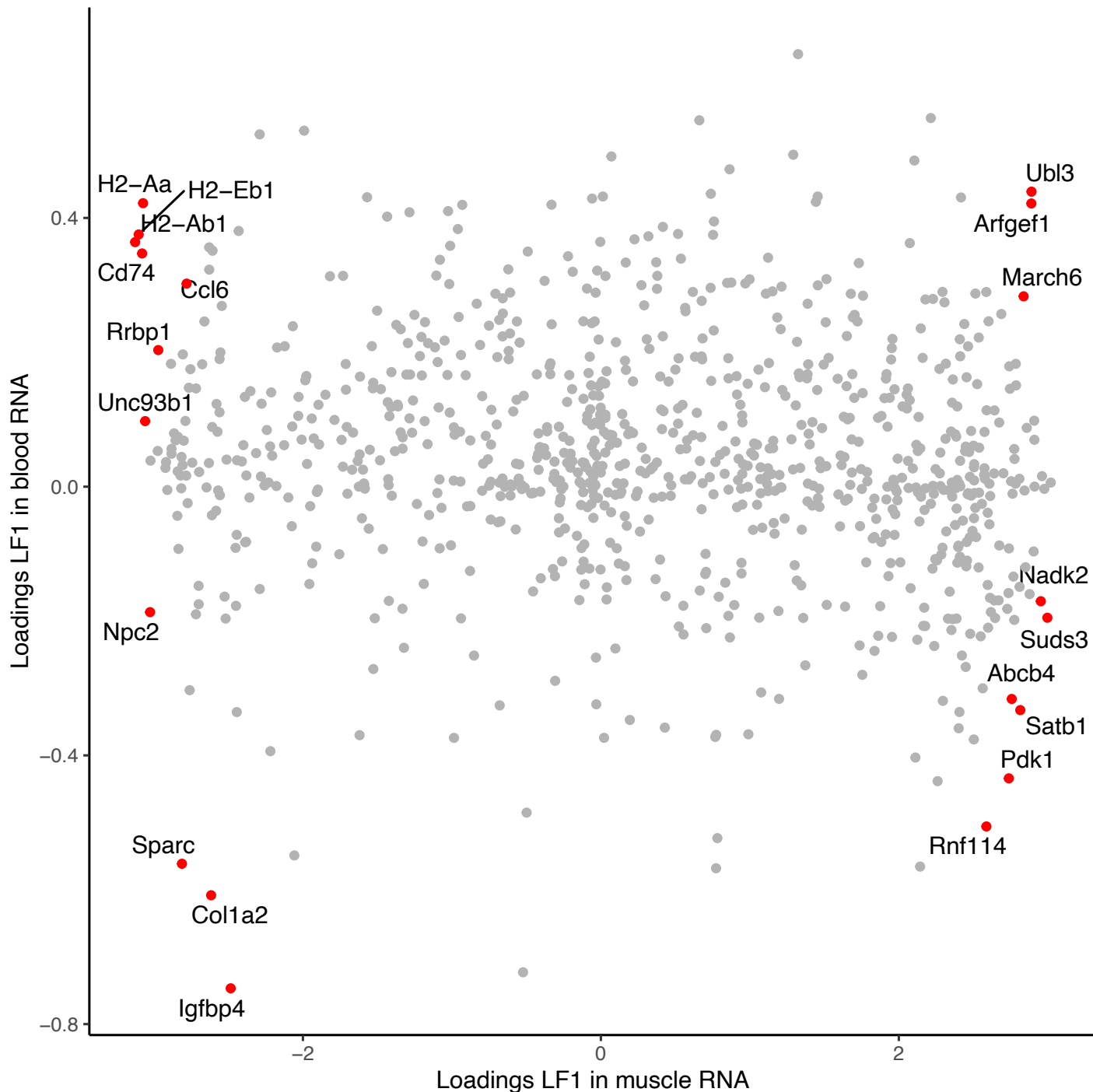

Supplementary Figure 3

Supplement: S8 Fig — (PDF) [file pone.0283869.s008.pdf]
