# Supplementary material for: Multiomic characterization of disease progression in mice lacking dystrophin
Source: PLoS One. 2023 Mar 31;18(3):e0283869. doi: 10.1371/journal.pone.0283869 (PMC10065259; doi:10.1371/journal.pone.0283869)
Supplement: Attachment — Submitted filename: rebuttal.pdf [file pone.0283869.s014.pdf]

# POINT BY POINT REPLY TO THE COMMENTS FROM THE ACADEMIC EDITOR AND THE 4 REFEREES

**Manuscript:** Multiomic characterization of disease progression in mice lacking dystrophin

**Manuscript ID:** PONE-D-22-31031

## REPLY TO THE ACADEMIC EDITOR

**Comments from the AE:** Although the reviewers commented favorably on some aspects of your study manuscript, there were significant criticisms that preclude publication. Major weaknesses include 1) The pathological data of several models with several timing used in this study should be shown. 2) LF1 is strongly associated with mouse group and time. The details of LF1 should be shown such as regeneration factors and novel or unknown factors for muscle pathology.

**Reply:** we would like to thank PLOS ONE for the opportunity to submit a revised version of our manuscript and to reply to the constructive feedback from the 4 referees. In this document we provide with a point by point answer to the comments from the referees.
As concerns weakness (1), we have performed H&E staining and included representative images in Supplementary Figure 1 for all mouse models at two different magnifications (see also our reply to comment #1 from referee #1).
With respect to point (2), we have proceeded to a more careful assessment of regeneration factors associated with LF1 (see our answer to comment #2 from referee #1), and made an attempt to pinpoint potential unknown factors for muscle pathology (see our reply to comment #3 from referee #2).

**REPLY TO REFEREE # 1**

**Overall assessment:** In this article, they tried to find biomarkers for DMD by Multi-Omics Factor Analysis (MOFA) with skeletal muscle samples and blood samples of DMD model mice. They identified some latent factors (LF) to discriminate dystrophic and healthy mice. MOFA enabled to connect the gene expression in dystrophic muscles, pro-fibrotic and energy metabolism alteration. They clearly showed some factors from MOFA to connect gene expression analysis and metabolic analysis.

**Reply:** We thank the reviewer for the time spent to evaluate our manuscript and the positive feedback. Below we provide a reply to their 3 comments.

**Remark #1.** In their studies, authors used dystrophic skeletal muscle samples (TA) for Multi-Omics Factor Analysis (MOFA). Readers will want to know how the skeletal muscle pathology of model mice that they used for analysis are. They should show the skeletal muscle pathology (stained sections of TA with H&E stain etc) of mice that they used in their studies.

**Reply:** We have performed H&E staining as suggested by the reviewer. Representative images are now presented in Supplementary Figure 1 for all mouse models at two different magnifications.

**Remark #2.** They have identified some LFs which are able to discriminate dystrophic and healthy mice. They mentioned some molecules and genes related to fibrosis, regeneration and inflammation in LF1. Regarding regeneration factors, they should mention how the expression of typical myogenesis-related factors, myoD, myogenin, pax7 etc were changed and related to their analysis if they have any data.

**Reply:** We thank the reviewer for the suggestion to look into regeneration factors with more detail. In the previous version of the manuscript we mentioned that genes involved in muscle regeneration such as *Myl4*, *Mybph*, *Sparc*, *Gpx1* are represented in LF1; however, we did not mention the 3 genes suggested by the referee.

To answer this question we looked into whether *Myod1*, *Myog* and *Pax7* were found to be differentially expressed (DE) between WT and dystrophic mice in the muscle RNA-seq omic view, which we previously reported as single omic analysis (Signorelli *et al.*, 2021, dataset EV2); moreover, we checked how strongly these genes are associated with LF1.

*Myod1* and *Myog* were found to be DE between WT and *mdx* mice (BH adjusted p-values = 0.000030 and 0.002785, respectively) by Signorelli *et al.* (2021), whereas for P*ax7* there was not enough evidence of differential expression (BH adjusted p-value = 0.22). The same conclusion holds for the comparisons between WT and, respectively, *mdx++* and *mdx+-* mice.

Besides being DE in dystrophic mice, we see that *Myod1* and *Myog* are strongly associated with LF1: their factor loadings for LF1 are -2.429 and -2.440, very close to the 5-th percentile (-2.456) of the distribution of all loadings (in absolute value) for LF1 in muscle (Supplementary File 1). *Pax7* was not included in the training of MOFA, as it was not among the top 2500 expressed genes in muscle on which MOFA was trained.

To conclude, both *Myod1* and *Myog* are DE in dystrophic mice, and at the same time they are strongly associated with LF1. We have therefore added them among the list of genes (mentioned in Section: "Multiomic dystrophic signature (LF1)") that are well-represented in LF1 and involved in muscle regeneration.

**Reference:** Signorelli, M., Ebrahimpoor, M. et al. (2021). Peripheral blood transcriptome profiling enables monitoring disease progression in dystrophic mice and patients. *EMBO Molecular Medicine*, 13, e13328.

**Remark #3.** They mentioned that there were Col6a1, Col6a2, Col6a3 genes in LF1 in their result. Ullrich muscular dystrophy (MD) is known to be a different MD from DMD, which caused by abnormal collagen VI, extracellular matrix. In this studies, they analyzed samples of DMD model mice, lack of dystrophin. They should mention that how their results are related to two different MDs.

**Reply:** In the previous manuscript version we reported that Col6a1, Col6a2 and Col6a3 are among the top 100 genes by factor loading in LF1. These genes are responsible for Bethlem myopathy and Ullrich muscular dystrophy. Pathogenic variants in these genes can cause the disease with both recessive and dominant inheritance patters. Recessive variants (homozygous or compound heterozygous) lead to large reduction or absence of collagen 6, while dominant forms affect packaging of the collagen 6 monomers into dimers, tetramers and microfibrils.

We have looked into these genes further, and found out that they are all similarly elevated in dystrophic mice, as shown in the figure below (also included as Supplementary Figure 5 in the revised manuscript):

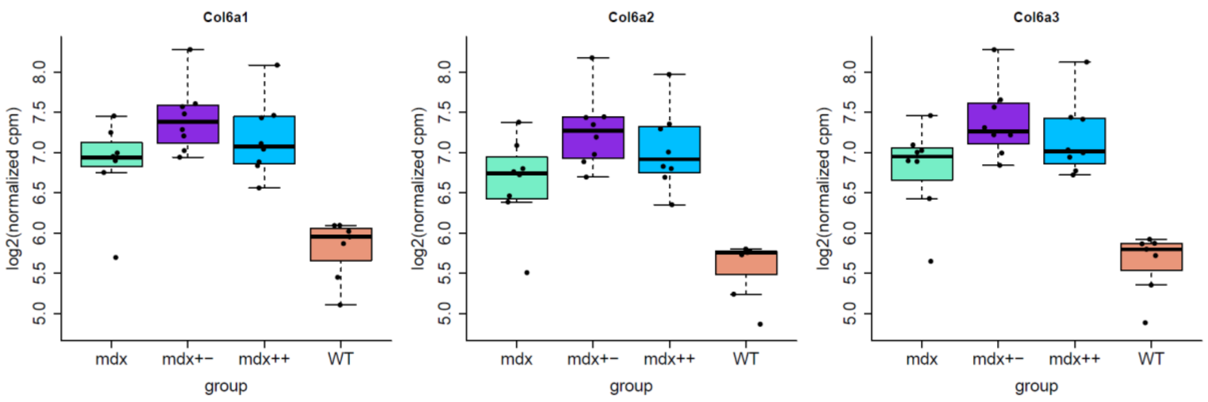

Since we observe an increase of collagen 6 expression, we believe that our findings are not related to the mechanism observed in recessively inherited collagen 6 myopathies. Given that no mutations are present in collagen 6 genes in the mice studied in this manuscript, we believe that no effect on microfibrils formation can be expected either, suggesting that the pathogenic mechanism observed in dominant collagen 6 myopathies does not apply in our context. We therefore hypothesize that the increased expression of collagen 6 genes is mirroring the increase in extracellular matrix due to the fibrosis present in mdx mice.

It would be interesting to follow-up on this points raised by the reviewer, and compare the signature we identified in mdx mice to the signature of Collagen 6 deficient mice. However, at the moment we cannot directly compare our results across the two diseases as we did not perform a multi-omic study in collagen 6 deficient mice, and we are not aware of publicly-available data that could be used to perform this comparison. This is something that could be done in prospective studies.

We would also like to note that multiple genes causing neuromuscular disorders showed high loading in LF1. This point is further discussed in the answer to remark 3 raised by referee 3.

**REPLY TO REFEREE # 2**

**Overall assessment:** The submitted manuscript entitled “Multiomic characterization of disease progression in mice lacking dystrophin” by Signorelli et al., reported the results of MOFA for disease and age dependent difference in mdx mice models using muscle and blood RNA-seq, blood lipidomics and metabolomics. It is very interesting study to understand the comprehensive signatures of the pathogenesis in mdx mice. The authors found that disease dependent signature as LF1 and age dependent signatures as LF4 and LF5. Especially in LF1, various known genes and molecules were included. It is noteworthy to mention that some gene expression of enzymes as well as their catabolites were simultaneously included. Although this reviewer can agree this manuscript for further process, there are some concerns as below.

**Reply:** We thank the reviewer for the useful comments about our work. Hereafter we provide an answer to their 3 comments.

**Remark #1.** While this study is important to understand the pathophysiological features of mdx mice, there is no information about human muscular dystrophy. The authors should analyze LF1, 4 or 5 with registered human datasets.

**Reply:** we thank the reviewer for the interesting suggestion. One of the advantages of MOFA is that it estimates latent factors whose computation simply involves a linear combination of the (normalized) values of molecules across different omic views. However, a major limitation of this approach is that to be able to do such computation for a new dataset, one needs to gather measures of all the omic views on which the original MOFA model was trained. In the case of our model, that would require joint availability of longitudinal blood RNA-seq, lipids and metabolites, as well as of cross-sectional muscle RNA-seq. Unfortunately, we are not aware of a multiomic dataset on dystrophic individuals that would satisfy these features, and we are thus unable to directly apply the fitted model to human datasets.

Although such a multiomic comparison between mouse and human is not possible, we would like to point out that a single-omic comparison is more straightforward and possible. As a matter of fact, we have performed such comparison for blood RNA-seq in a previously published manuscript (Signorelli *et al.*, 2021) where we considered both the blood data from the mouse experiment and blood RNA-seq data from healthy individuals and dystrophic patients. In that comparison, we showed that many of the genes found to be DE in *mdx* mice were also DE in a cohort of 39 dystrophic patients. Moreover, we have added to the revised manuscript a comparison of the list of genes more strongly associated with LF1 to information on human muscular dystrophies as explained in our answer to remark #3 (see below).

**Reference:** Signorelli, M., Ebrahimpoor, M. et al. (2021). Peripheral blood transcriptome profiling enables monitoring disease progression in dystrophic mice and patients. *EMBO Molecular Medicine*, 13, e13328.

**Remark #2.** In addition, this strategy seems very powerful to understand the entire pathogenesis of mdx mice model, however, there is no information about other models or diseases. The authors should discuss at this point.

**Reply:** we thank the reviewer for the suggestion. We indeed agree that the proposed approach is powerful to study the relationship across tissues and time points, and as such it would provide evidence for converging pathological mechanisms across different neuromuscular disease. We did not include information about other models and diseases as we do not have phased multi-omic data available across blood and muscle for other models and diseases at this stage. We are also not aware of other studies for which multi-omic data would be readily available. It is worth mentioning that performing such studies for other diseases require 7 months long study and expensive multi-omic data acquisition and analyses. While this would be quite interesting to do, we think that the results of this study could be used by other groups to select a subset of markers to be explored in other models and diseases. We have added 2 sentences to the Discussion section suggesting to perform such studies in other models and diseases.

**Remark #3.** Especially in LF1, there are well-known genes or molecules, which are related to skeletal muscle pathology. How about novel or unknown gene(s) for muscle pathology among LF1? If there is such genes, the authors must discuss about the relationship between genes and muscle. If not, the authors must describe at this point.

**Reply:** To answer this question we selected the genes with high loadings (absolute value > 2) in LF1. This produced a list of 883 mouse genes. The mouse gene IDs were converted into human gene IDs using Biomart, reducing the number to 840 genes. Such list was then and compared to the list of genes known to cause neuromuscular disorders, which we obtained from Benarroch et al. (2020); for completeness, we took the online table available at https://www.musclegenetable.fr/index.html.

82 genes with high loading in LF1 are known to cause neuromuscular conditions. These include collagen 6 genes (responsible for Bethlem myopathy and Ullrich muscular dystrophy, please see also response to remark 3 by referee 1), but also Ryr1, Cav3, Lama2 and Ttn among others. Supplementary File 3 lists all genes with high loadings in LF1 that relate to neuromuscular conditions, and has been added to the revised manuscript.

For the remaining 758 genes, we looked whether association with human muscular phenotype exist using the Euretos database. Euretos collects information from literature and databases in the form of a knowledge graph. We found evidence that another 257 genes were previously related to muscular phenotype. Examples were the Atad1 gene, which was previously

associated with muscle stiffness (Wolf et al., 2018) and Tmem70 previously associated with severe neonatal muscular hypotonia (Honzik et al., 2010). After filtering these 257 genes, we still had a list of 501 genes that showed high loading in LF1. This new piece of analysis has now been added to the manuscript and the list of 501 genes is now provided as Supplementary File 4.

We have added these additional analyses to the Section “Multiomic dystrophic signature (LF1).

**References:**

Benarroch, L., Bonne, G., et al. (2020). The 2021 version of the gene table of neuromuscular disorders (nuclear genome). *Neuromuscular Disorders*, 30(12), 1008-1048.

Honzík, T., Tesařová, M., et al. (2010). Mitochondrial encephalocardio-myopathy with early neonatal onset due to TMEM70 mutation. Archives of disease in childhood, 95(4), 296-301.

Wolf, N. I., Zschocke, J., et al. (2018). ATAD1 encephalopathy and stiff baby syndrome: a recognizable clinical presentation. Brain, 141(6), e49-e49.

**REPLY TO REFEREE # 3**

**Overall assessment:** This paper attempts to identify factors that correlate with the progression of DMD with age in mdx mice and in mdx+/- and mdx+/+ mice by means of a multi-omics analysis. There were no major problems with the strategy used in this study, and the experiments were conducted logically. However, there are some points that remain unclear, which should be clarified by the authors.

**Reply:** We thank the reviewer for the positive evaluation of our manuscript and constructive feedback. Hereafter is our answer to their 2 comments.

**Remark #1.** It is not clear what is the purpose of using *mdx+/-* and *mdx+/+* in addition to *mdx* in the first place. In addition, the results and discussion do not seem to clearly mention the benefits obtained by adding these two mice.

**Reply:** we thank the reviewer for giving us the opportunity to clarify the reasons why we included *mdx +/+* and *+/-* mice in our experiment. The inclusion of these mice was decided based on a previous report that showed how *mdx+/-* mice performed significantly worse compared to *mdx+/+* mice (van Putten et al, 2012). When designing the study, we therefore decided to include *mdx+/-* and *mdx+/+* to be able to compare them and check whether we could detect a signature associated with severity. The data presented in our manuscritpt and in our previous RNA-seq analyses (Signorelli et al, 2021) do not offer evidence of a more severe signature in *mdx utrn+/-* mice compared to *mdx utrn+/+*, suggesting that either our approach is not sensitive enough to capture such differences or that the differences between these mouse models are not as clear as previously anticipated. This part has now been added to the Discussion.

**References:**

Signorelli, M., Ebrahimpoor, M. et al. (2021). Peripheral blood transcriptome profiling enables monitoring disease progression in dystrophic mice and patients. *EMBO Molecular Medicine*, 13, e13328.

van Putten, M., Kumar, D., et al. (2012). Comparison of skeletal muscle pathology and motor function of dystrophin and utrophin deficient mouse strains. *Neuromuscular Disorders,* 22, 406 – 417.

**Remark #2.** Conclusions are unclear. It is necessary to clearly state which of the factors involved in the pathological progression of DMD identified in this study could be used as useful markers in the future.

**Reply:** We understand this comment as a request to narrow down the markers to be used in future studies. A shortlist would indeed help other investigators to focus on a few for validation studies for example. Even though we could attempt to select a few biomarkers of

interest, in our opinion such a selection would be arbitrary, as it would be based on a multiomic approach that exploits correlation across biomarkers, rather than on the previously reported analyses of differential expression (Signorelli et al., 2021; Tsonaka et al., 2020; Tsonaka et al., 2021) that provide more direct evidence of the association between individual molecules and phenotypes.

Rather than trying to pinpoint individual markers of disease progression (which was one of the goal of the aforementioned previous studies), in this manuscript we attempt to identify how genes, metabolites and lipids relate to each other based on the inferred multi-omic latent factors. We believe our analysis, which is not intended to prioritize among genes based on loadings, matches this objective. Should we select based on factor loadings, we would probably provide a more pleasing, but less scientifically solid answer. We are, however, trying to answer this question in follow-up work by relating such profiles to histological alterations by spatial analysis approaches (Heezen et al., 2022). Through this approach, we aim to narrow down the list and provide a clearer context of use for the multiomic associations discovered in this study.

**References:**

Heezen, L.G.M., Abdelaal, T. et al. (2022). Spatial transcriptomics reveal markers of histopathological changes in Duchenne muscular dystrophy mouse models. *bioRxiv* 2022.03.17.484699

Signorelli, M., Ebrahimpoor, M. et al. (2021). Peripheral blood transcriptome profiling enables monitoring disease progression in dystrophic mice and patients. *EMBO Molecular Medicine*, 13, e13328.

Tsonaka R, Signorelli M, et al. (2020). Longitudinal metabolomic analysis of plasma enables modeling disease progression in Duchenne muscular dystrophy mouse models. *Human Molecular Genetics*, 29: 745–755.

Tsonaka R, Seyer A, Aartsma-Rus A, Spitali P. (2021). Plasma lipidomic analysis shows a disease progression signature in *mdx* mice. *Scientific Reports*, 11: 12993.

**REPLY TO REFEREE # 4**

**Overall assessment:** In this study, the authors conducted a multiomic analysis of longitudinal trajectories by tissue in DMD mice and identified biomarkers that could observe the etiology and progression of DMD through MOFA analysis. In particular, by identifying pro-fibrotic and energy metabolism alterations, inflammation and lipid signatures that can reveal muscle conditions in the blood, it presents meaningful results in the diagnosis and treatment of DMD.

This study was conducted in a timely manner to understand the pathogenesis, diagnosis and treatment of DMD. In addition, by analyzing multiomic readouts obtained from longitudinal/across tissues in a genius way, it contributed greatly to understanding the cause and progression of disease through a multiomic approach.

This study is a great inspiration for DMD and muscle disease researchers, and is worthy enough to be published with the following minor modifications.

**Reply:** we would like to thank the reviewer for the very positive assessment our manuscript. Below we provide an answer to their 6 comments.

**Remark #1.** (Line 127) Briefly summarize and describe the method of preparation and acquisition of the metabolite and lipids levels.

**Reply:** thank you for the comment. We have added a summary of the preparation and acquisition of metabolites and lipids to the manuscript. For more details, we refer readers to the articles of Tsonaka et al. (2020) and Tsonaka et al. (2021) where the metabolite and lipid data were presented and analysed separately (in a single-omic, rather than multiomic, fashion).

**References:**

Tsonaka R, Signorelli M, et al. (2020). Longitudinal metabolomic analysis of plasma enables modeling disease progression in Duchenne muscular dystrophy mouse models. *Human Molecular Genetics*, 29: 745–755.

Tsonaka R, Seyer A, Aartsma-Rus A, Spitali P. (2021). Plasma lipidomic analysis shows a disease progression signature in *mdx* mice. *Scientific Reports*, 11: 12993.

**Remark #2.** (Line 133) Consistently describe the variable m as a superscript or subscript in the method.

**Reply:** Thank you for pointing out this formatting issue. We have fixed it in the new manuscript version.

**Remark #3.** (Line 265) Represent the missing E in Figure 2.

**Reply:** Thank you for pointing out this formatting issue. We have fixed it in the new version of Figure 2.

**Remark #4.** For non-expert understanding, please provide a graphical summary of MOFA, model training and selection method applied in this study.

**Reply:** we thank the reviewer for the suggestion. Although we believe that illustrating how MOFA works is not the main goal of this article since the model was introduced and described by Argelaguet et al. (2018), we have nevertheless made an attempt to provide a graphical representation of how MOFA makes it possible to decompose the data in each view into a matrix of view-specific weights and a matrix of shared latent factors. This image is now included as Supplementary Figure 2 in our revised manuscript, and it was inspired by Figure 1 of Arguelaguet et al. (2018)'s article.

**Reference:** Argelaguet, R., Velten, B., Arnol, D., Dietrich, S., Zenz, T., Marioni, J. C., ... & Stegle, O. (2018). Multi-Omics Factor Analysis - a framework for unsupervised integration of multi-omics data sets. *Molecular Systems Biology*, 14(6), e8124.

**Remark #5.** Please describe the full name other than the symbol of the gene.

**Reply:** we thank the reviewer for this suggestion. We have considered whether to add full gene names to both the manuscript and all figures and supplementary files. However, we came to the conclusion that this would make the article and figures less readable as full gene names can be quite long. Therefore, we have decided to continue to refer to genes through their gene symbols, as customary in the field.

**Remark #6.** Using biochemical methods in mouse tissues, please determine the levels of RNAs, metabolites, and lipids involved in fibrosis, muscle regeneration, and immune responses that represent LF1.

**Reply:** We thank the reviewer for this suggestion. We interpret this point as a request to validate the genes, metabolites and lipids identified in this study in relationship with fibrosis, regeneration and immune response. Given the large numbers of features identified across omic views, we cannot commit to validate hundreds of features with a second technology such as qPCR for genes related to regeneration, inflammation and fibrosis. We would however like to point out we are currently using spatial transcriptomics to relate such findings in relation to histological alterations in mice. Spatial analysis of gene expression allows to study a large number of genes in histological sections and relate their expression to histological findings. The work is currently under review, however a preprint is already available in bioRxiv (Heezen et al, 2022). As an example we refer to figure 3 of this paper,

where we indeed show that genes such as Myl4 and Sparc that show high loading in LF1 are indeed associated with areas of intensive regeneration in *mdx* mice.

**References:**

Heezen, L.G.M., Abdelaal, T. et al. (2022). Spatial transcriptomics reveal markers of histopathological changes in Duchenne muscular dystrophy mouse models. *bioRxiv* 2022.03.17.484699
